# Inhibitors in AKTion: ATP-competitive vs allosteric

**DOI:** 10.1042/BST20190777

**Published:** 2020-05-26

**Authors:** Glorianne Lazaro, Eleftherios Kostaras, Igor Vivanco

**Affiliations:** Division of Cancer Therapeutics, The Institute of Cancer Research, 15 Cotswold Rd., SM2 5NG London, U.K.

**Keywords:** AKT, allosteric, ATP-competitive, cancer, inhibitors

## Abstract

Aberrant activation of the PI3K pathway is one of the commonest oncogenic events in human cancer. AKT is a key mediator of PI3K oncogenic function, and thus has been intensely pursued as a therapeutic target. Multiple AKT inhibitors, broadly classified as either ATP-competitive or allosteric, are currently in various stages of clinical development. Herein, we review the evidence for AKT dependence in human tumours and focus on its therapeutic targeting by the two drug classes. We highlight the future prospects for the development and implementation of more effective context-specific AKT inhibitors aided by our increasing knowledge of both its regulation and some previously unrecognised non-canonical functions.

## Introduction

AKT is a family of serine/threonine kinases consisting of three isoforms (AKT1, AKT2 and AKT3), regulated upstream by the activation of phosphatidylinositol 3-kinases (PI3K) following growth factor stimulation. Several downstream substrates of activated AKT play a major role in the regulation of cell size, cell cycle progression, glucose metabolism, genome stability, transcription, protein synthesis and inhibition of pro-apoptotic proteins [1–4]. Dysregulation of AKT-dependent pathways is associated with the development and maintenance of various solid tumours such as those of the lung, prostate, endometrium, cervix, skin and breast [5–7]. Given the role of AKT as a critical signalling hub for tumour survival, significant efforts have been made to target this kinase for many years.

Two major classes of small-molecule AKT inhibitors, namely allosteric and ATP-competitive, have entered clinical development (see Table 1) based on strong pre-clinical evidence of anti-tumour activity [8]. The allosteric inhibitor MK-2206, the earliest selective AKT inhibitor to reach the clinic, showed promising evidence of AKT signalling blockade and tolerability in its first-in-man trial [9]. The initial enthusiasm about AKT inhibitors as therapeutics, however, began to dissipate after limited clinical activity was observed for many compounds in various early phase studies. In fact, until very recently, no AKT inhibitor either alone or in combination had reached Phase III trials. A likely factor contributing to the disappointing clinical performance of these drugs is the paucity of robust predictive biomarkers for patient stratification. But, significant progress has been made recently with the implementation of precision medicine platforms to stratify patients based on genotype. In particular, two basket studies, MSK-IMPACT (actively recruiting) and the NCI molecular analysis for therapy choice (NCI-MATCH) trial, in which patients with AKT-mutant tumours were treated with the ATP-competitive AKT inhibitor AZD5363 (capivasertib) have shown encouraging data with response rates of ∼25% [10]. However, many questions remain regarding the factors that limit the size of the responsive patient population, and whether the performance of individual compounds can be better matched to specific genotypes and/or histologies.

**Table 1.** AKT inhibitors in the clinic

Drug	Company	Class	Target	Clinical development (monotherapy)	Clinical development (combination)
AZD5363	Astra Zeneca	ATP-competitive	AKT1/2/3, P70S6K/PKA	**Phase I** MSK-IMPACT — solid tumours with molecular match mutations (A) Prostate cancer (C) **Phase II** NCI-MATCH — advanced solid tumours, lymphomas or multiple myeloma with molecular match mutations (A) NSCLC (A) ER+ breast cancer vs placebo (C) Advanced breast cancer (A)	**Phase I/II** **Enzalutamide** — prostate, mCRPC (A) **Paclitaxel** — ER+ breast cancer, TNBC (A), mBC (A), gastric cancer (T) **Abiraterone** — mCRPC (A) **Docetaxel and prednisolone (DP)** — mCRPC (A) **Olaparib** — advanced tumours (C), various gynaecological tumours (A) **Olaparib **+** durvalumab** — advanced tumours (A) **Fulvestrant** — ER+ advanced breast cancer (A)
Ipatasertib/GDC-0068	Roche	ATP-competitive	AKT1/2/3	**Phase I** Solid tumours (C) **Phase II** Glioblastoma (U) NCI-MATCH — advanced solid tumours, lymphomas or multiple myeloma with molecular match mutations (A)	**Phase I/II** **GDC-0973** — solid tumours (C) **Paclitaxel** — breast cancer and other solid tumours (C) **Docetaxel** — solid tumours (A) **Trastuzumab and pertuzumab** — breast cancer (A) **Fulvestrant, aromatase inhibitor, and palbociclib** — breast cancer (A) **mFOLFOX6 (oxaliplatin, leucovorin, 5FU)** — gastric cancer and other solid tumours (A) **Enzalutamide** — gastric cancer and other solid tumours (A) **Abiraterone** — prostate cancer (A) **Rucaparin** — breast, ovarian and prostate cancer (A) **Carboplatin and paclitaxel** — breast cancer (A) **Phase 1b/III** **Palbociclib and fulvestrant** — breast cancer (A)
GSK690693	Glaxosmithkline	ATP-competitive	AKT1/2/3, PKA, PrkX, PKC	**Phase I** Solid tumours or lymphoma (T) Haematologic malignancies (T)	None
GSK2141795	Glaxosmithkline	ATP-competitive	AKT1/2/3	**Phase I** Solid tumours or lymphoma (C) Ovarian cancer (C)	**Phase I/II** **Dabrafenib and trametinib** — solid tumours with BRAF mutation (S) **Trametinib** — cervical cancer (T), BRAF WT melanoma (C), AML (T), mTNBC (C), myeloma (A), uveal melanoma (C), endometrial cancer (A) **GSK1120212** — TNBC and BRAF-WT melanoma (C)
GSK2110183	Glaxosmithkline	ATP-competitive	AKT1/2/3	**Phase I** Healthy volunteers (C) Haematological malignancies (C) Multiple myeloma (T) **Phase I/II** Solid tumours and Haematologic malignancies (C) Langerhans cell histiocytosis (C)	**Phase I/II** **GSK1120212** — multiple myeloma and solid tumours (C) **Carboplatin and paclitaxel** — ovarian cancer (C) **Bortezomib and dexamethasone** — multiple myeloma (C) **Ofatumumab** — CLL (C)
LY2780301	Lilly	ATP-competitive	AKT1/2/3 p70S6 kinase	**Phase I** Solid tumours, non-Hodgkin's lymphoma (C)	**Phase I/II** **Gemcitabine** — solid tumours and non-Hodgkin's lymphoma (C) **Paclitaxel** — HER2+ breast tumours (T)
MK-2206	Merck	Allosteric	AKT1/2	**Phase I** Advanced and metastatic solid tumours (C), leukaemia (C) **Phase II** Platinum-resistant ovarian, fallopian tube or peritoneal cancer (C) Adenoid cyst carcinoma (C) Head and neck cancer (C) Endometrial cancer (C) Breast cancer (T) Lymphoma (C) AML (C) Advanced liver cancer (T) Metastatic or locally advanced colon and renal cancer (C) Diffuse large B cell lymphoma (T) Advanced gastric or gastroesophageal junction cancer (C) Metastatic neuroendocrine tumours (C) Biliary cancer (C) Refractory kidney cancer (T) Recurent nasopharyngea carcinoma (C) Lung cancer and thymic malignancies (A)	**Phase I/II** **Gefitinib** — NSCLC (U) **Hydroxychloroquine** — advanced solid tumours, melanoma, prostate or kidney cancer (A) **Trastuzumab** — HER2+ breast cancer (A) **Trastuzumab + lapatinib** — HER2+ breast cancer, gastric or gastrointestinal cancers, solid tumours (T) **Trastuzumab + paclitaxel** — HER2+ solid tumours (T) **Lapatinib** — advanced metastatic tumours or breast cancer (C) **Paclitaxel** — solid tumours, breast cancer (C) **Carboplatin **+ **paclitaxel**, or **docetaxel**, or **erlotinib** — advanced or metastatic solid tumours **Anastrazole **+ **fulvestrant** — mBC (C) **Ridaforolimus** — advanced tumours (C) **Dalotuzumab** — advanced tumours (T) **Selumetinib** — advanced tumours (C), colorectal cancer (C), melanoma (T), pancreatic cancer (C), NSCLC (A) **Erlotinib** — NSCLC (C) **Dinaciclib** — pancreatic cancer (C) **Goserelin acetate** — breast cancer (T) **Bicalutamide** — prostate cancer (A) **Bendamustine **+ **rituximab** — CLL or small lymphocytic leukaemia (C)
ARQ 092	Arqule/Merck	Allosteric	AKT1/2/3	**Phase I** Adults with proteus syndrome, PIK3CA-related overgrowth spectrum (PROS) (A) Solid tumours, lymphoma (C)	**Phase I/II** **Carboplatin **±** paclitaxel** — solid tumours (A) **Anastrozole** — ovarian and endometrial cancer (A)
ARQ 751	Arqule/Merck	Allosteric	PanAKT	**Phase I** Solid tumours with PIK3CA/AKT/PTEN mutations (A)	**Phase I** **Fulvestrant** — breast cancer (A) **Paclitaxel** — solid tumours (A)
BAY1125976	Bayer	Allosteric	AKT1/2	**Phase I** Solid tumours (C)	None

**Development status**: Active (A) — the study is ongoing, and participants are receiving an intervention or being examined, Completed (C) — the study has ended normally, and participants are no longer being examined, Suspended (S) — the study temporarily stopped for assessment, Terminated (T) — the study has stopped early and will not start again, Unknown (U) — status not known. **Other**: mCRPC, metastatic castration-resistant prostate cancer; TNBC, triple negative breast cancer; mBC, metastatic breast cancer; NSCLC, non-small cell lung cancer; CLL, chronic lymphocytic leukaemia; AML, acute myeloid leukaemia.

In this review, we will discuss the evidence that supports a role for AKT in tumour maintenance and the molecular complexity of AKT dependence in cancer cells. Importantly, we review the different classes of AKT inhibitors, their efficacy and distinct mechanisms of action, as well as how these properties can be used to improve patient stratification.

## Targeting AKT pathway activation as a therapeutic strategy

**Figure 1.**
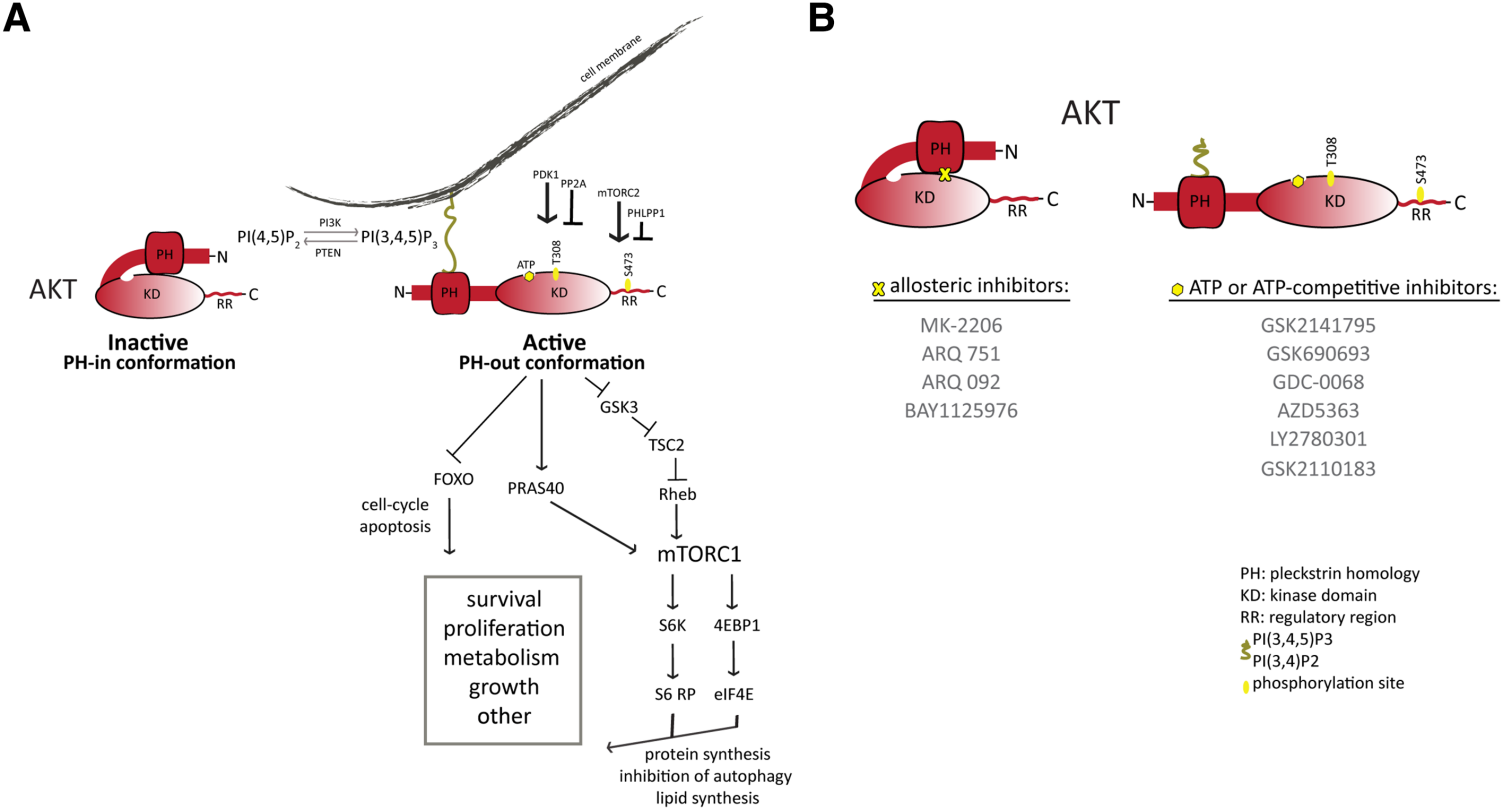
Activation and inhibition of AKT. (**A**) PtdIns(3,4,5)P3 (PIP3) is maintained at the cell surface by the opposing function of several class I PI3K kinases and the PTEN phosphatase. AKT remains inactive in the cytoplasm in a PH-in conformation facilitated by interactions of the PH and the kinase domains. Membrane anchored AKT acquires a PH-out conformation, becomes phosphorylated at specific residues by specific kinases and subsequently propagates the signal by phosphorylating different effectors such as PRAS40, GSK3b, FOXO and more. AKT activation increases protein and lipid synthesis, whereas inhibits autophagy and cell apoptosis. (**B**) Differential mechanism of action of allosteric and ATP-competitive inhibitors of AKT. Allosteric inhibitors lock the PH-in conformation and suppress its membrane localisation and activation, whereas ATP-competitive inhibitors bind to the ATP-pocket of the kinase domain, stabilise the PH-out conformation where AKT becomes phosphorylated and increase its membrane localisation.

All three AKT proteins have three functional domains: an N-terminal fragment with a pleckstrin-homology (PH) domain, a central kinase domain (KD) and a C-terminal fragment with a regulatory region (RR) containing a hydrophobic motif (Figure 1). Under basal unstimulated conditions, AKT sits in the cytoplasm in an inactive conformation (PH-in) maintained by intramolecular interactions between the PH and KDs. AKT activation is initiated at the plasma membrane by the action of receptor tyrosine kinases (RTKs), G-protein-coupled receptors (GPCR) or small Ras-related GTPases that turn on class I PI3K kinases driving the synthesis of the phosphoinositide second messenger PI(3,4,5)P3 (or PIP3). Binding of PIP3 (or its dephosphorylated product PI(3,4)P2) to the PH domain of AKT relocalises AKT to the plasma membrane causing destabilisation of the auto-inhibited conformation and driving allosteric activation by promoting high-affinity substrate binding [11]. This new de-inhibited conformation (PH-out) exposes the kinase and regulatory domains making them accessible to regulatory kinases that phosphorylate AKT1 at two key residues [12–14]. mTORC2 and PDK1 phosphorylate S473 in the RR hydrophobic motif and T308 in the activation loop of the KD, respectively, leading to full activation of AKT1 kinase (Figure 1A). Similarly, AKT2 and AKT3 are activated through dual phosphorylation on corresponding residues (T309 and S474 on AKT2, and T305 and S472 on AKT3) [15–18]. This molecular understanding of how AKT is activated along with current structural information have aided in deciphering the mechanisms of action of various small-molecule AKT inhibitors.

As discussed above, AKT functions as a signal transducer by phosphorylating many protein substrates that contain the minimal consensus recognition motif of R-X-R-X-X-S/T-φ (where X is any amino acid and φ a hydrophobic residue) [3]. Many of these proteins regulate cellular functions associated with tumour initiation and/or maintenance [3]. Consequently, the main objective in drug development has been to generate compounds capable of inhibiting AKT-mediated phosphorylation. Such efforts have led to several clinical AKT inhibitors which can be classified into two classes based on their mode of inhibition, either ATP-competitive or allosteric (Figure 1B). The former target the catalytic site of the active kinase in the PH-out conformation and prevent substrate phosphorylation [19,20], whilst the latter target an allosteric pocket within the PH-domain/kinase-domain interface that stabilises the PH-in conformation [12,13,21]. Allosteric AKT inhibitors, therefore, lock AKT in an auto-inhibited conformation and interfere with PH-domain mediated-membrane recruitment, thus preventing AKT kinase activation and AKT phosphorylation [21]. Accordingly, binding of allosteric AKT inhibitors to AKT cause a decrease in AKT regulatory site phosphorylation and AKT substrate phosphorylation in cells. Interestingly, as long as AKT remains bound to PIP3 or PI(3,4)P2, ATP-binding site occupancy by either ATP-competitive AKT inhibitors or ATP (but not ADP) leads to a paradoxical increase in AKT phosphorylation on both S473 and T308 [14]. This is thought to be caused by enhanced plasma membrane localisation and a shielding ‘cage’ conformation stabilised by intramolecular interactions between Arg 273 and His 194 that protects against dephosphorylation by PP2A and PHLPP1 phosphatases [20,22,23]. The biological consequences of AKT hyperphosphorylation in an otherwise kinase-inactive AKT molecule (i.e. ATP-competitive-inhibitor-bound AKT) in cells with increased PI3K signalling (and therefore increased basal levels of PIP3) have not been investigated. However, given that many kinases, including AKT, have been reported to have non-catalytic functions [23] (some of which are dependent on AKT phosphorylation state [24]), it is possible that these biochemical changes are not entirely inconsequential.

In addition to the two major classes of AKT inhibitors described herein, Weisner et al. recently described the development of borussertib, a first-in-class covalent-allosteric AKT inhibitor. This compound irreversibly binds to two non-catalytic cysteines in AKT (at positions 296 and 310) located in an interdomain pocket between the PH and KD thereby stabilising the PH-in conformation. It has shown anti-proliferative activity *in vitro* and in PDX models when combined with the MEK inhibitor trametinib [25,26]. Although this class of compounds is yet to be tested clinically, their improved residence time could provide increased efficacy through more favourable tumour pharmacokinetics.

## Susceptibility to AKT inhibitors as a function of AKT activation mechanism and cellular context

Aberrant activation of AKT can result from a variety of genetic lesions that target either AKT directly, components of the PI3K pathway upstream of AKT, or negative regulators of PI3K signalling. These lesions include amplification and/or mutations in RTK genes (e.g. EGFR or HER2), PDK1 or PIK3CA (one of the catalytic subunits of PI3K), or loss of function mutations, deletions, or epigenetic silencing of tumour suppressor genes such as PTEN or INPP4B which oppose PI3K signalling by dephosphorylating its lipid products. Whether and how specific AKT-activating lesions can influence the extent to which tumours will become dependent on AKT-driven signalling (or sensitive to AKT inhibitors) is poorly understood. This type of knowledge will be critically important in improving patient stratification.

The existing pre-clinical evidence indeed suggests that different AKT-activating lesions can lead to distinct biochemical and biological consequences and each can result in different susceptibilities to AKT inhibitors. For example, using quantitative mass spectrometry, Moniz et al. [27] found that homozygous PTEN inactivation led to changes in the phospho-proteome that were clearly distinct from those caused by an activating PIK3CA mutation (H1047R). Another study found that the transcriptional targets of PI3K activation in immortalised lung epithelial cells differ significantly depending on the activating lesion (i.e. AKT/PIK3CA/PTEN), and showed that only a few transcripts were affected similarly by all lesions examined, further highlighting the complexity of the network [28]. *In vivo*, two studies evaluated the anti-tumour activity of either ipatasertib or capivasertib (both ATP-competitive AKT inhibitors) found that although these drugs showed growth suppression in a range of human xenograft models with AKT-activating lesions, regressions were more commonly observed in tumours with homozygous PTEN inactivation compared with those with activating PIK3CA mutations [29,30].

## Consequence of AKT mutations to AKT inhibitor sensitivity

Activating somatic mutations in AKT occur at very low frequency in multiple cancer types. The most common of these mutations, E17K, targets a glutamic acid in the PH domain which causes enhanced membrane association, constitutive AKT activation, and has been shown to be transforming *in vitro* [31]. In wild type AKT, E17 forms a salt bridge with R273 in the KD, supporting the stability of the PH-in conformation. This interaction is lost in the E17K mutant, causing a shift in the conformational equilibrium towards the PH-out conformation which exposes the ATP-binding site and consequently favours binding of ATP-competitive inhibitors [21]. Consistently, *in vitro* kinase assays showed that the AKT1-E17K mutant had increased sensitivity to ipatasertib and capivasertib but decreased sensitivity to the allosteric Inhibitor AKT inhibitor VIII. Interestingly, while the allosteric inhibitor MK-2206 predictably shows marginal inhibition of AKT1-E17K, two other allosteric AKT inhibitors, ARQ 092 and ARQ 751, have demonstrated potency against this mutant, excellent anti-tumour activity both in cell lines and PDX models [32], and promising results in early phase studies, including in a patient with an E17K mutation [33].

This suggests that additional determinants of allosteric inhibitor binding can affect their ability to target this mutant. The fact that patients whose tumours carry an AKT1-E17K mutation have shown high response rates following treatment with the ATP-competitive-inhibitor-capivasertib suggests that, in a subset of patients, this mechanism of AKT activation can render tumours susceptible to AKT kinase inhibition. The frequency and clonality of this mutation in tumour specimens were also shown to correlate with capivasertib response in two Phase I studies [34,35]. However, together with the fact that not all AKT-mutant tumours were responsive to AKT inhibition, these data suggest that additional contextual information (i.e. biomarkers) will be needed to better predict when the AKT1-E17K mutation can identify responsive tumours.

Albeit at much lower frequency, additional activating mutations in AKT genes have been reported [36]. These include AKT1 L52R, Q79K and D323H, all of which weaken interdomain (PH-KD) interactions, increase in-cell kinase activity and promote cell survival [37]. Similar to E17K, these mutants remained sensitive to ATP-competitive inhibitors, but are less sensitive to allosteric inhibitors (including MK-2206) [11,14,38]. Thus, understanding the impact of individual AKT-activating mutations on the response to AKT inhibitors will likely require a systematic evaluation of multiple AKT inhibitors of each class.

## Targeting non-catalytic AKT functions

In addition to the well-documented kinase-dependent functions of AKT, there is evidence that AKT can regulate some cellular processes independently of enzymatic activity. Remy et al. [24] reported that binding of AKT to SMAD3 can inhibit SMAD3-mediated transcription and protect cells against TGF-β-induced apoptosis, an effect that does not require AKT kinase activity.

Vivanco et al. have shown that the catalytic activity of AKT is dispensable for cancer cell survival in certain contexts (e.g. in breast cancer cells with concurrent activating PIK3CA mutations and HER2 gene amplification, or lung cancer cells with MET gene amplification). This work showed that ectopic expression of kinase-deficient AKT alleles (K179M AKT1, K181M AKT2, G161V AKT2) can protect cells from cell death induced by an allosteric AKT inhibitor [39]. It also showed that although ATP-competitive and allosteric AKT inhibitors can potently suppress cell proliferation in AKT-dependent cells, significant induction of cell death was only seen with the latter, despite more significant inhibition of kinase activity with the former. This increased sensitivity to allosteric AKT inhibitors suggests that this class of drugs may be able to target, at least partly, non-catalytic AKT functions and could, therefore, represent a better therapeutic option in some tumours with constitutively increased AKT-dependent signalling due to abnormally high cellular levels of PIP3 or PI(3,4)P2 caused by PI3K-pathway-activating mutations.

## Isoform-specific targeting of AKT

All ATP-competitive inhibitors in clinical development are able to target (albeit with variable potency) all three AKT isoforms (i.e. AKT1, AKT2 and AKT3), while allosteric inhibitors generally spare AKT3 [40]. This distinction is of significant therapeutic relevance in melanoma and in subsets of triple-negative breast cancers, where AKT activation occurs primarily through AKT3 gene amplification [40–43]. Furthermore, RNAi-mediated silencing of AKT3 has been shown to promote the growth of human vascular tumours *in vivo*, and to accelerate their growth *in vitro* [44]. However, in the same tumours, AKT1 silencing had growth-suppressive effects. Therefore, while ATP-competitive inhibitors might be useful in cases where AKT3 is constitutively activated and acts as an oncogenic driver, allosteric AKT3-sparing compounds might be preferable in tumours where AKT3 has been shown to have growth inhibitory properties. In addition to AKT3, AKT2 has also been found to have tumour suppressive functions in certain tissues as shown in two separate mouse models of breast cancer, where AKT2 deletion accelerated tumour development while AKT1 deletion inhibited tumour growth [45]. These data raise the question of whether isoform-selective AKT inhibitors could avoid the potential collateral damage that pan-AKT inhibitors might cause in tumours where specific AKT isoforms may be tumour suppressive. Recently, Quambusch et al. [46] used a structure-based approach to identify isoform-specific residues surrounding the allosteric pockets of AKT isoforms to guide the design of isoform-selective covalent-allosteric AKT inhibitors. Such compounds can be very useful in assessing isoform-specific contributions to AKT dependence in tumours, and could lead to the generation of drugs that provide a wider therapeutic window.

## AKT post-translational modifications

AKT is also known to undergo an array of post-translational modifications (PTMs) in addition to phosphorylation that modulate its activity, stability and localisation [47]. For example, growth-factor-induced K63-linked ubiquitination within the PH domain of AKT triggers membrane localisation and kinase activation [48,49]. SUMOylation has also been reported to modulate AKT phosphorylation [50,51] and kinase activity [52] and can regulate G1/S cell cycle transition and Bcl-x pre-mRNA splicing [53]. Recently, two studies showed that SETDB1-mediated AKT methylation can promote AKT phosphorylation and activation [54,55]. The impact of AKT inhibitor binding on PTMs (beyond phosphorylation of the regulatory sites) and of PTMs on AKT inhibitor binding has not been thoroughly investigated. But, given the potential impact of these modifications on the biophysical properties of AKT, a closer look is warranted.

## AKT inhibitors in the clinic

The following allosteric AKT inhibitors have been tested or are currently being tested in phase I/II trials (Table 1): MK-2206, ARQ 092, ARQ 751 and BAY1125976. MK-2206, ARQ 092 and ARQ 751 have demonstrated positive safety profiles [9,56,57]. Phase II monotherapy trials of MK-2206 have shown limited clinical activity in many tumour types [58–62]. Of note, one of these studies [58] showed that despite pharmacodynamic evidence of target inhibition in surrogate tissues, there was no significant inhibition of AKT in the tumours of patients treated with tolerable doses of MK-2206, suggesting that the lack of clinical benefit could be partly due to suboptimal pharmacology. ARQ 092 has also been tested in both solid and haematopoietic malignancies and has demonstrated acceptable tolerability, though with limited activity and a few partial responses [33,56]. Finally, ARQ 751 is currently being tested in a phase 1 study for solid tumours with PIK3CA/AKT/PTEN mutations (NCT02761694) both as monotherapy and in combination with other anti-cancer agents.

Multiple ATP-competitive AKT inhibitors have also undergone clinical testing (Table 1). These include GSK2141795, GSK690693 and LY2780301, all of which were tested in phase I studies [63–65]. Similar to allosteric inhibitors, the anti-tumour activity of these compounds was not significant, despite favourable safety profiles that suggest off-target effects inherent to ATP-competitive agents are unlikely to be problematic. AZD5363 and GDC-0068 have been tested in several monotherapy trials. In one study, AZD5363 showed anti-tumour activity in 50% of patients with PIK3CA-mutant tumours, however, the magnitude of the effects was not significant enough to warrant further monotherapy testing [58]. GDC-0068 has shown some anti-tumour activity (30% stable disease) across tumour types in a phase I study [66].

Because AKT inhibitor monotherapy has failed to show significant therapeutic benefit, the clinical focus has shifted towards combination strategies. There is ample pre-clinical rationale for AKT inhibitor combination treatments in various settings including acquired resistance to other anti-cancer agents, adaptive *de novo* resistance due to relief of negative feedback, and both chemo- and radio-resistance. In all these scenarios, the evidence suggests that AKT activation is implicated in these mechanisms of resistance. Therefore, AKT inhibitors are currently being tested in combination with many chemo-therapeutic and other targeted agents. Those showing promising therapeutic activity include GDC-0068 which has been combined with abiraterone, a CYP17A1 inhibitor, to treat prostate cancer patients with PTEN loss and results to date show improved progression free survival (PFS) and overall survival (OS) [67]. GDC-0068 is also currently being tested in combination with paclitaxel in patients with PIK3CA/AKT1/PTEN-Altered breast tumours (NCT03337724). AZD5363 has recently been tested in a randomised trial in combination with fulvestrant in postmenopausal women with ER+/HER2− negative [68] and in combination with paclitaxel in triple-negative breast tumours [69], both studies showing significantly longer PFS and an improvement in OS.

Finally, the most encouraging clinical data with AKT inhibitor monotherapy comes from the NCI-MATCH trial, a molecularly stratified phase II basket study. According to the most recent update, 35 AKT1-E17K-positive patients had been recruited within the AZD5363 sub-protocol, and the overall response rate in these patients was reported to be 25% [10].

## Conclusions and perspectives

1. Conceptually, AKT continues to be a highly attractive therapeutic target because of the multiplicity of oncogenic functions it regulates, and because as a kinase, it represents a very druggable target class. However, the translation of AKT inhibition into therapeutic benefit has been more complicated than initially anticipated. The complexity and numerous layers of AKT regulation hinder our ability to accurately predict the biochemical and biological consequences of AKT inhibition. To understand the biological activity of AKT-targeting agents, the molecular details of these complexities need to be understood in various contexts which will be defined by tissue of origin, tumour type, and the specific genotype that drives AKT activation. With perhaps the exception of the AKT1-E17K mutation, there are no robust predictive biomarkers of AKT inhibitor response. Importantly, this mutation can predict response to most ATP-competitive inhibitors, but only to select allosteric inhibitors, which highlights the need to understand the impact of mutation on binding to the specific targeting agent. Multiple context-specific biomarkers will likely be required in order to define appropriate patient populations that might benefit from specific AKT inhibitors.
2. It is also important to consider what aspect of AKT function needs to be targeted in a given context for maximum therapeutic benefit, as growing evidence that the non-catalytic functions of AKT can also play a role in tumour maintenance. This consideration will likely influence the choice of AKT inhibitor, as ATP-competitive inhibitors do not appear to significantly impact non-enzymatic AKT activities. Allosteric inhibitors, on the other hand, seem to partly inhibit non-catalytic AKT functions, although optimisation of these compounds to enhance this property may be required to improve their efficacy. To enable optimisation and development of next-generation AKT inhibitors that can target catalytic and non-catalytic functions more efficiently, further work will be required to characterise the molecular identity of non-catalytic downstream effectors that can serve as pharmacodynamics biomarkers.
3. Target engagement can be significantly influenced by drug-specific and drug-class-specific differences in isoform and conformation selectivity, and by the effects of mutation on the accessibility to drug binding sites. Activating mutations such as AKT1-E17K or AKT1-D223H, for example, can destabilise the PH-in conformation and therefore confer resistance to allosteric AKT inhibitors but sensitivity to ATP-competitive inhibitors. Therefore, while certain genotypes could render tumour cells AKT-dependent, they could also make them resistant to certain AKT inhibitors. Similarly, should acquired resistance to AKT inhibitors occur through AKT gene mutations, it is likely that the nature of the mutations will be driven by the binding properties of the inhibitor. In this context, the availability of different classes of inhibitors with unique binding properties could, therefore, offer the possibility of second-line treatments where the efficacy of some existing drugs may not be affected by specific mutations. But, given the reported failure of MK-2206 to effectively inhibit AKT in breast tumours, it is important to not only consider different AKT inhibitors for different contexts, but also explore alternative dosing schedules that may help to overcome pharmacokinetic limitations.

## Perspectives

*Importance of the field*: AKT is a critical PI3K effector kinase involved in a variety of oncogenic processes, but therapeutic targeting of AKT in cancer has had modest clinical success in single agent strategies.
*Summary of current thinking*: Two main classes of small molecule AKT inhibitors (ATP competitive and allosteric) are in clinical development, both of which could be useful in specific contexts.
*Future directions*: Detailed understanding of both the biology of AKT and how each inhibitor class influences various aspects of AKT-regulated processes in genotype-defined contexts is critical to maximise therapeutic benefit.

## Abbreviations

KD: kinase domain
OS: overall survival
PFS: progression free survival
PH: pleckstrin-homology
PI3K: phosphatidylinositol 3-kinases
PTMs: post-translational modifications
RR: regulatory region
RTKs: receptor tyrosine kinases
